# Supplementary material for: Cyclophosphamide Induces Lipid and Metabolite Perturbation in Amniotic Fluid during Rat Embryonic Development
Source: Metabolites. 2022 Nov 12;12(11):1105. doi: 10.3390/metabo12111105 (PMC9693482; doi:10.3390/metabo12111105)
Supplement: Supplementary file 1 [file metabolites-12-01105-s001.zip › metabolites-1970211-supplementary.pdf]

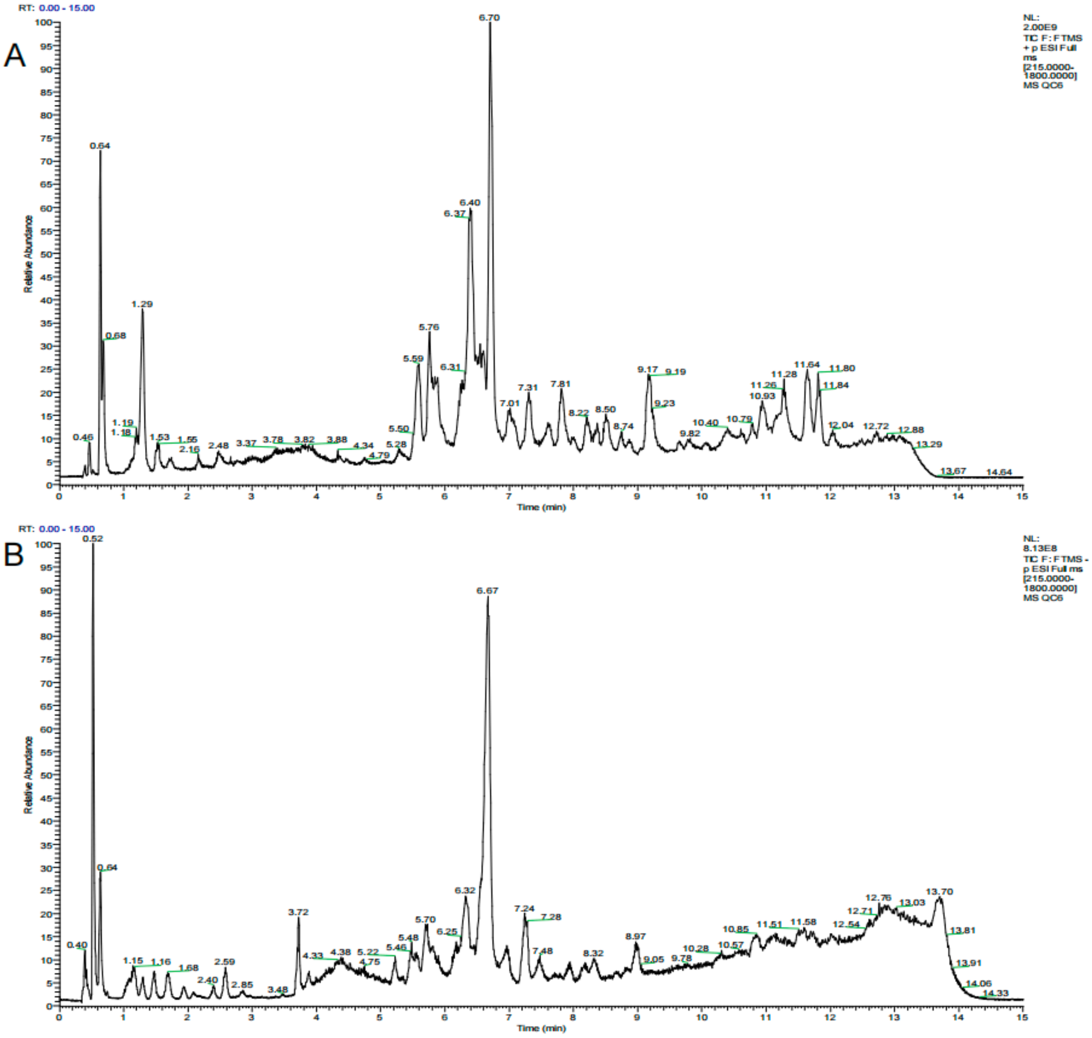

Figure S1. TICs of lipids from rat AF of QC samples in MS-DIAL software. (A) positive ion mode; (B) negative ion mode.

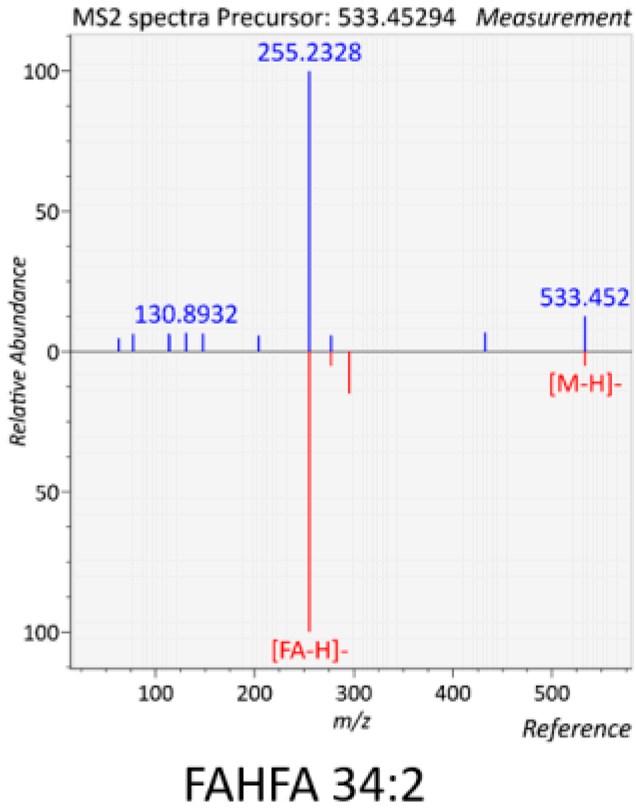

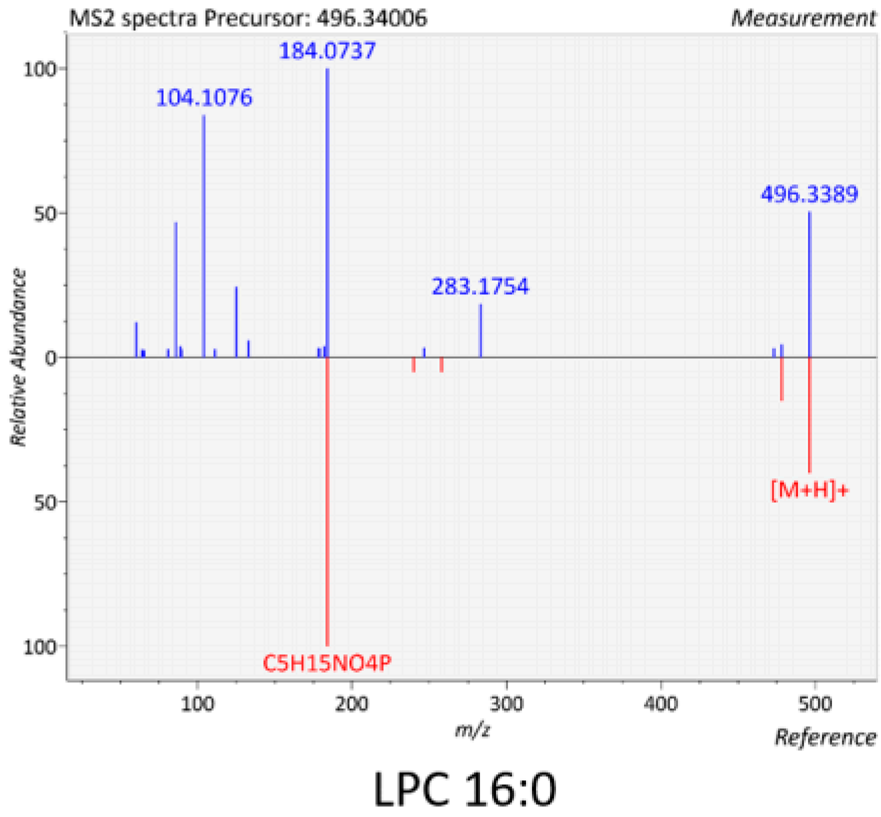

Figure S2. Selected lipids with matching fragmentation patterns to the LipidBlast library with MS-DIAL software

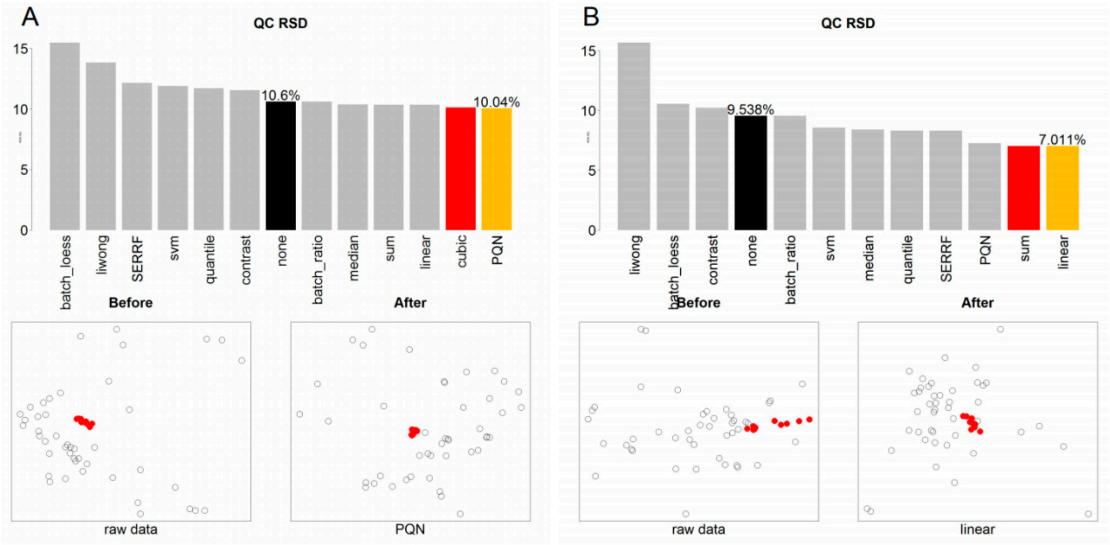

Figure S3. RSD values in QC samples obtained by different normalization methods (A) positive ion mode; (B) negative ion mode.

Table S1. Differential lipids in AF (positive ion mode)

Significant metabolites	m/z	Rt (min)	Adduction	PPM mass error	P value	FDR	FC (CK/CP)
CAR 20:4	448.3406	1.41	[M]+	−3. 469	0.026	0.17	0.79
CAR 24:0	512.4688	4.91	[M]+	2. 877	0.002	0.08	0.67
CE 24:5	744.6622	11.43	$[M+NH_4]+$	−4. 213	0.026	0.17	0.58
Cer 42:0;2O	652.6613	9.23	[M+H]+	1. 575	0.002	0.08	1.31
Cer 42:1;2O	650.6406	8.60	[M+H]+	−6. 074	0.002	0.08	1.35
DG O-33:1	584.5611	6.55	$[M+NH_4]+$	−0. 233	0.004	0.08	1.29
DG O-35:1	612.5910	7.25	$[M+NH_4]+$	−2. 508	0.004	0.08	1.24
DG O-37:1	640.6216	7.94	$[M+NH_4]+$	−3. 429	0.004	0.08	1.25
LPC 16:1	516.3032	1.09	[M+Na]+	−2. 369	0.041	0.21	1.35
LPC 16:1	494.3228	1.09	[M+H]+	−2. 763	0.026	0.17	1.27
LPC 18:3	518.3226	0.98	[M+H]+	−2. 866	0.009	0.10	1.34
LPC 19:0	538.3870	3.06	[M+H]+	0. 565	0.015	0.13	0.46
LPC 20:2	548.3702	1.61	[M+H]+	−1. 506	0.004	0.08	1.43
LPC 20:3	546.3530	1.64	[M+H]+	−4. 367	0.002	0.08	1.56
PC 40:8	830.5715	5.26	[M+H]+	2. 527	0.004	0.08	0.80
PC O-40:5	822.6428	6.81	[M+H]+	6. 933	0.015	0.13	1.44
PI 38:6	900.5494	4.92	$[M+NH_4]+$	−11. 409	0.009	0.10	1.81
PS 38:3	814.5660	5.97	[M+H]+	8. 249	0.004	0.08	1.44
SM 35:1;2O	717.5852	5.85	[M+H]+	−7. 374	0.026	0.17	0.71
TG 46:0	796.7399	12.75	$[M+NH_4]+$	0. 03	0.009	0.10	1.51
TG 48:0	824.7693	12.81	$[M+NH_4]+$	−1. 062	0.004	0.08	1.35
TG 48:3	818.7253	10.46	$[M+NH_4]+$	2. 521	0.026	0.17	1.22
TG 51:4	858.7478	10.87	$[M+NH_4]+$	−7. 821	0.015	0.13	0.53
TG 53:0	894.8464	12.46	$[M+NH_4]+$	−2. 209	0.041	0.21	1.30
TG 53:6	882.7594	10.50	$[M+NH_4]+$	5. 532	0.015	0.13	0.64
TG 54:0	908.8567	12.58	$[M+NH_4]+$	−8. 116	0.009	0.10	1.35
TG 54:0	908.8691	12.57	$[M+NH_4]+$	5. 582	0.009	0.10	1.30
TG 54:5	898.7811	11.12	$[M+NH_4]+$	−5. 214	0.026	0.17	0.82
TG 54:5	903.7454	11.12	[M+Na]+	4. 59	0.026	0.17	0.82
TG 55:0	922.8779	12.64	$[M+NH_4]+$	−1. 936	0.004	0.08	1.31
TG 55:1	920.8617	12.48	$[M+NH_4]+$	−2. 581	0.041	0.21	1.37
TG 55:4	914.8077	11.64	$[M+NH_4]+$	−10. 25	0.009	0.10	0.32
TG 55:5	912.8046	11.34	$[M+NH_4]+$	3. 4	0.009	0.10	0.60
TG 56:0	936.8996	12.71	$[M+NH_4]+$	4. 518	0.015	0.13	1.30
TG 56:0	936.8893	12.72	$[M+NH_4]+$	−6. 433	0.002	0.08	1.28
TG 56:10	916.7410	9.76	$[M+NH_4]+$	2. 295	0.002	0.08	0.36
TG 56:6	924.7965	11.18	[M+NH4]+	−5. 359	0.041	0.21	0.81
TG 56:8	925.7293	10.36	[M+Na]+	−4. 049	0.041	0.21	0.82
TG 57:0	950.9137	12.77	$[M+NH_4]+$	2. 822	0.009	0.10	1.22

TG 57:1	948.9003	12.65	[M+NH4]+	5. 231	0.015	0.13	1.50
TG 57:7	936.7937	11.17	[M+NH4]+	−8. 29	0.026	0.17	0.45
TG 59:1	976.9228	12.77	[M+NH4]+	−3. 968	0.026	0.17	1.24
TG 60:1	990.9410	12.82	[M+NH4]+	−1. 349	0.041	0.21	1.20
TG 60:5	982.8842	12.11	[M+NH4]+	4. 521	0.015	0.13	0.69
TG 60:7	978.8496	11.60	[M+NH4]+	1. 158	0.041	0.21	0.82

Table S2. Differential lipids in AF (negative ion mode)

Significant metabolites	m/z	Rt (min)	Adduction	PPM mass error	p value	FDR	FC (CK/CP)
FA 22:2	335.2962	4.00	[M-H]-	2. 017	0.026	0.13	0.56
FA 22:4	331.2630	2.85	[M-H]-	−3. 694	0.002	0.04	0.62
FA 22:5	329.2481	2.26	[M-H]-	−1. 499	0.002	0.04	0.56
FA 24:4	359.2942	3.76	[M-H]-	−3. 796	0.002	0.04	0.49
FA 24:5	357.28091	3.281	[M-H]-	2. 817	0.002	0.04	0.62
FA 26:1	393.3745	5.81	[M-H]-	1. 795	0.015	0.09	0.54
FA 26:5	385.3100	3.98	[M-H]-	−3. 124	0.002	0.04	0.35
LPC 17:0	568.3639	1.89	[M+CH3COO]-	3. 339	0.009	0.07	0.67
LPC 22:0	638.4384	4.21	[M+CH3COO]-	−2. 964	0.045	0.17	0.45
LPE O-16:0	438.3000	1.80	[M-H]-	2. 264	0.041	0.16	0.36
PC O-34:2	802.59784	6.58	[M+CH3COO]-	1. 367	0.015	0.09	0.39
PC O-38:4	854.6299	6.74	[M+CH3COO]-	2. 15	0.041	0.16	0.60
PC O-39:5	866.6317	5.99	[M+CH3COO]-	−3. 731	0.009	0.07	0.57
PE O-42:7	802.5765	7.20	[M-H]-	1. 154	0.041	0.16	0.52
PI 35:2	847.5294	4.87	[M-H]-	−5. 713	0.041	0.16	0.60
PI 37:4	871.5330	4.97	[M-H]-	−1. 356	0.041	0.16	0.50
PI 40:4	913.5756	5.59	[M-H]-	−6. 121	0.041	0.16	0.34
SM 35:1;2O	775.5988	5.81	[M+CH3COO]-	2. 247	0.004	0.05	0.61
SM 36:1;2O	775.6005	6.14	[M+HCOO]-	4. 361	0.002	0.04	0.53
SM 38:2;2O	815.6271	6.25	[M+CH3COO]-	−1. 591	0.004	0.05	0.56
SM 40:2;2O	843.6611	6.92	[M+CH3COO]-	1. 651	0.009	0.07	0.65
SM 40:3;2O	841.6415	6.27	[M+CH3COO]-	−3. 027	0.002	0.04	0.61

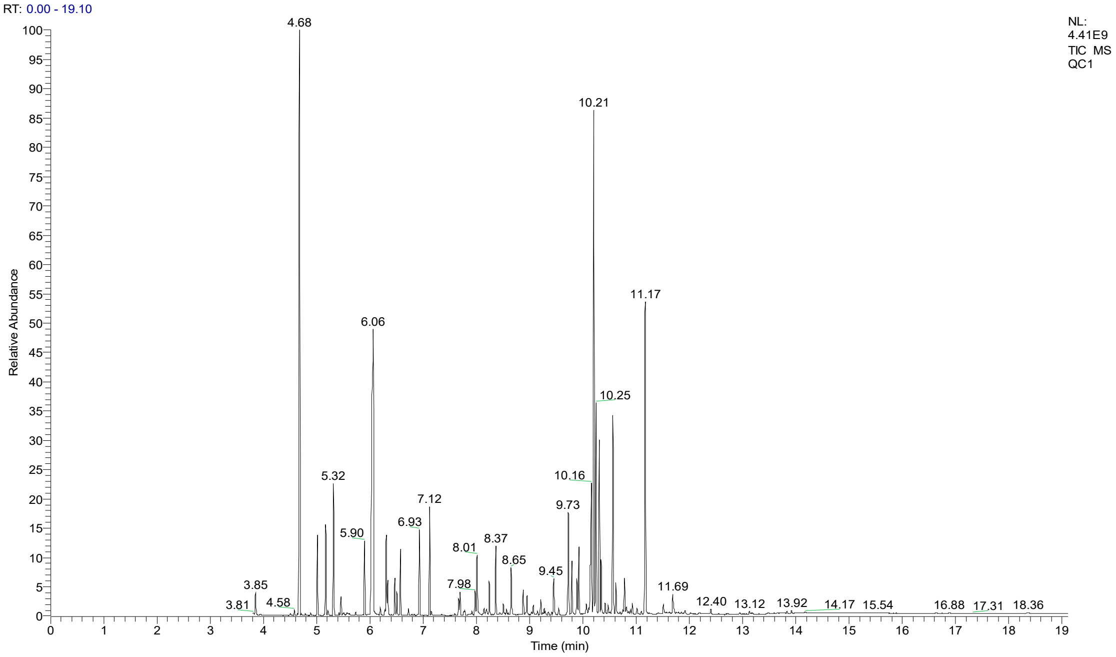

Figure S4. TICs of metabolites from rat AF of QC samples in GC-MS experiments.

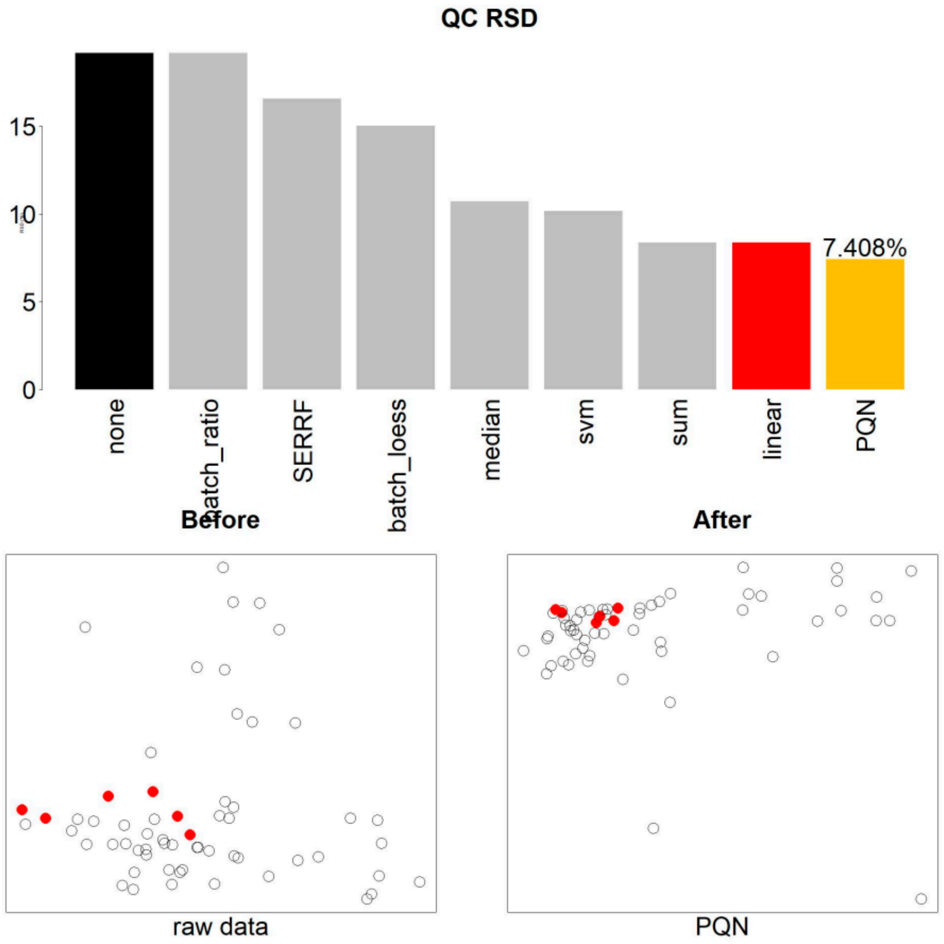

Figure S5. RSD values in QC samples obtained by different normalization methods in GC-MS experiments.

Table S3. Differential metabolites of AF detected in GC-MS

Metabolite name	FC（CK/CP）	p value	FDR	HMDB	PubChem	KEGG
stearic acid	1.64	0.000	0.049	HMDB0000827	5281	C01530
palmitic acid	1.60	0.000	0.049	HMDB0000220	985	C00249
tryptophan	1.54	0.009	0.155	HMDB0000929	6305	C00078
glycerol-1-phosphate	0.59	0.005	0.109	HMDB0000126	439162	C00093
